# Nutritional and Phytochemical Characterization of Commercially Available Chia, Quinoa, Pumpkin Seed, Flaxseed and Triticale Products

**DOI:** 10.3390/plants15132079

**Published:** 2026-07-03

**Authors:** Eleni Giotaki, Valentina Perri, Nicholas J. Vaughan, Gary J. Duncan, Donna Henderson, Gary A. Cameron, Louise Cantlay, Jodie Park, Nicosha De Souza, Vassilios Raikos, Wendy R. Russell, Madalina Neacsu

**Affiliations:** 1 Rowett Institute, University of Aberdeen, Foresterhill, Aberdeen AB25 2ZD, UK; 2 Biomathematics and Statistics Scotland, Aberdeen AB25 2ZD, UK; 3 Department of Nutrition and Dietetics Sciences, School of Health Sciences, Hellenic Mediterranean University, 72300 Siteia, Greece

**Keywords:** dietary diversity, plant-based foods, nutritional composition, phytochemical profiling, sustainable diets

## Abstract

Limited data exists on the combined nutritional and phytochemical profiles of UK commercially available plant-based foods, limiting comprehensive compositional data available for dietary assessment and food formulation. This study addresses this gap by providing thorough compositional analysis of quinoa (red, black, organic), chia seeds (organic, white), pumpkin seeds (conventional, organic), flaxseeds (brown, golden, organic), and triticale grain (organic, cereal meal, rolled), profiling macronutrients, dietary fiber, amino acids, fatty acids, essential minerals, and bioactive phytochemicals. Pumpkin seeds exhibited the highest protein (29–36%) and fat (42–46%) contents, markedly exceeding quinoa and triticale, highlighting their role as a plant-based protein and energy source. Flaxseeds and chia seeds provided the greatest dietary fiber (15 g/100 g), while mineral analysis identified pumpkin seeds as particularly rich in phosphorus and magnesium, and white chia seeds as a rich source of calcium and iron. Targeted LC-MS/MS and HPLC screening (171 molecules) revealed substantial variation in phytochemical composition among products with red quinoa, golden flaxseed, and white chia seed containing the highest concentrations of quantified phytochemicals (up to 97.2 mg/100 g). These findings provide integrated data on the nutrient and phytochemical composition of selected commercially available products, reinforcing the practical importance of crop diversity for enhancing dietary nutrient and phytochemical diversity and informing future research, food innovation, and dietary assessment initiatives involving plant-based foods.

## 1. Introduction

The global burden of noncommunicable diseases (NCDs), including obesity, cardiovascular disease, type 2 diabetes, and certain cancers, continues to rise, largely driven by poor dietary patterns. The Western diet, characterized by high intakes of saturated fats, refined sugars, sodium, and ultra-processed foods, and low in dietary fiber, essential micronutrients, and phytochemicals, is strongly linked to this trend [1]. In contrast, plant-based and whole-food diets, rich in complex carbohydrates, unsaturated fats, fiber, and phytochemicals, are consistently associated with improved metabolic health, reduced inflammation, and lower environmental impacts compared with conventional dietary patterns [2].

A growing body of evidence highlights the importance of not only the quantity but also the quality and diversity of macronutrients in the diet. High-quality plant-based proteins, such as those found in seeds, legumes, and whole grains, provide essential amino acids and are often accompanied by dietary fiber, unsaturated fats, and bioactive peptides that support gut health and immune function [3]. Unlike many animal-based proteins, plant proteins are typically lower in saturated fat and cholesterol, and their consumption is associated with a reduced risk of cardiovascular and metabolic diseases [4].

Dietary fats also vary widely in their health effects. While saturated and trans fats are linked to increased cardiovascular risk, polyunsaturated fatty acids (PUFAs) and monounsaturated fatty acids (MUFAs), abundant in flaxseed, chia, and pumpkin seeds, have been shown to improve lipid profiles, reduce systemic inflammation, and support brain and cardiovascular health [5]. Of particular importance is the omega-6-to-omega-3 fatty acid ratio, which influences inflammatory pathways and overall metabolic balance. Western diets often exhibit a skewed ratio (up to 20:1), favoring omega-6 fatty acids, which can promote inflammation. In contrast, a more balanced ratio (ideally between 2:1 and 4:1) is associated with reduced risk of chronic diseases and improved cardiovascular outcomes [6].

Carbohydrate quality, especially in the form of dietary fiber, is critical in gut health. Fiber-rich foods such as seeds and whole grains provide non-starch polysaccharides that resist digestion in the upper gastrointestinal tract and are fermented by gut bacteria in the colon. This fermentation produces short-chain fatty acids (SCFAs) like butyrate, acetate, and propionate, which support gut barrier integrity, regulate immune responses, and reduce inflammation [7]. Recent clinical trials have shown that increasing the diversity of fiber-rich plant foods markedly improves gut microbiome composition, enhances microbial diversity, and reduces gastrointestinal symptoms [8].

Moreover, diverse sources of dietary fiber, including soluble, insoluble, and fermentable fiber, are essential for maintaining microbial diversity and metabolic flexibility. Fiber deprivation has been shown to reduce microbiota-derived B vitamins and impair immune regulation, while supplementation with specific fibers like inulin restores microbial function and immune homeostasis [9]. These findings underscore the importance of consuming not just adequate fiber, but a wide variety of fiber types from different plant sources to support optimal gut and immune health.

Mineral diversity is another cornerstone of nutritional adequacy. Many plant-based foods are rich in micronutrients such as magnesium, potassium, calcium, iron, and zinc, nutrients that are often under-consumed in Western populations. For example, chia seeds are especially high in calcium and iron, while pumpkin seeds are notable for their potassium content. These minerals are vital for bone health, oxygen transport, electrolyte balance, and enzymatic functions, and their adequate intake is linked to improved metabolic outcomes and reduced disease risk [10].

Beyond essential nutrients, phytochemicals, non-nutritive bioactive compounds found in plants, offer a wide range of health benefits. These include antioxidant, anti-inflammatory, antimicrobial, and cardioprotective effects. Compounds such as flavonoids, phenolic acids, lignans, and phytosterols have been shown to modulate oxidative stress, support vascular function, and influence gut microbiota composition [3]. Consequently, phytochemically rich foods such as quinoa, flaxseed, and chia may contribute substantially to dietary phytochemical intake. 

Importantly, dietary diversity also contributes to sustainable food systems. Encouraging the consumption of underutilized, nutrient-dense crops like triticale, quinoa, and pumpkin seeds may support diversification of food production systems and promote food security. [11]. These crops are often adaptable to diverse climates, require fewer inputs, and contribute to soil health and biodiversity. However, the environmental sustainability of specific food products depends on multiple factors, including production practices, supply chains, and geographical origin, which were not evaluated in the present study.

Pseudocereals and oilseeds, particularly quinoa, chia, and flaxseed, are widely distributed in the UK market, primarily driven by evolving patterns of agricultural diversification and supported by increasing consumer interest in plant-based nutrition and nutrient-rich dietary patterns. Selected nutrient-dense crops, including quinoa, chia, flaxseed, pumpkin seeds, and triticale, are commercially accessible through differentiated supply chains encompassing retail distribution, specialty functional-food markets, grain milling, and agricultural feed sectors [12]. Although commercially accessible within the UK agri-food system, quinoa, chia, flaxseed, pumpkin seeds, and triticale remain comparatively underexplored in terms of nutritional integration and dietary impact.

This study presents a thorough nutritional and phytochemical analysis of five plant-based crops: quinoa, chia, flaxseed, pumpkin, and triticale, commercially available in the UK. By evaluating their macronutrient composition, mineral content, fatty acid profiles, amino acid composition, and phytochemical richness, this study provides detailed compositional data for selected commercially available products. The findings are discussed in the context of dietary nutrient and phytochemical diversity. The aim of this study was to characterize and compare the nutritional and phytochemical composition of commercially available quinoa, chia, flaxseed, pumpkin seed, and triticale products available in the UK market.

## 2. Results

### 2.1. Overview of Nutritional Density and Diversity

**Figure 1:**
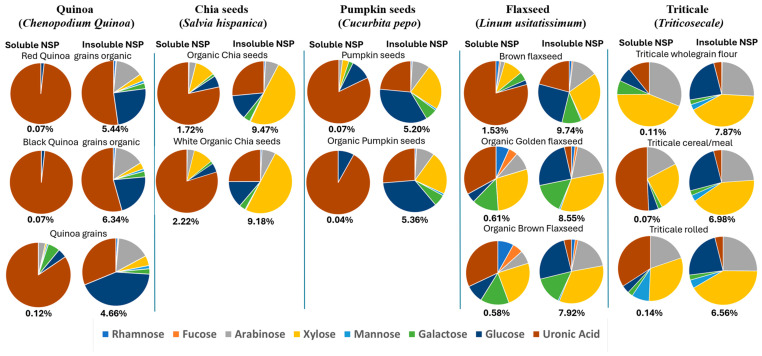
The distribution of monosaccharide composition of soluble non-starch polysaccharides (NSP) and insoluble NSP content in g per 100 g (average n = 3) milled crops for red quinoa grain organic, black quinoa grain organic, organic chia seeds, white organic chia seeds, pumpkin seeds, organic pumpkin seeds, brown flaxseed, organic golden flaxseed, organic brown flaxseed, triticale organic, triticale cereal/meal, triticale rolled.

**Figure 2:**
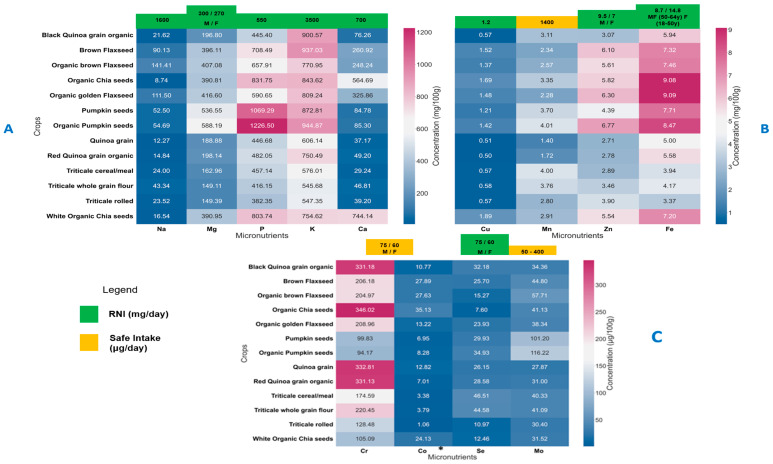
Heatmaps represent the average value of 3 independent measurements for each microelement in each crop using ICP-MS analysis The average values for Na, Mg, P, K, Ca along with the Reference Nutrient Intake (RNI) (**A**). The average values for Cu, Mn, Zn, Fe Micronutrients along with the Reference Nutrient Intake (RNI) (**B**). The average values for Cr, Co* Cobalt do not have RNI or Safe intake levels, Se, Mo (**C**).

**Figure 3:**
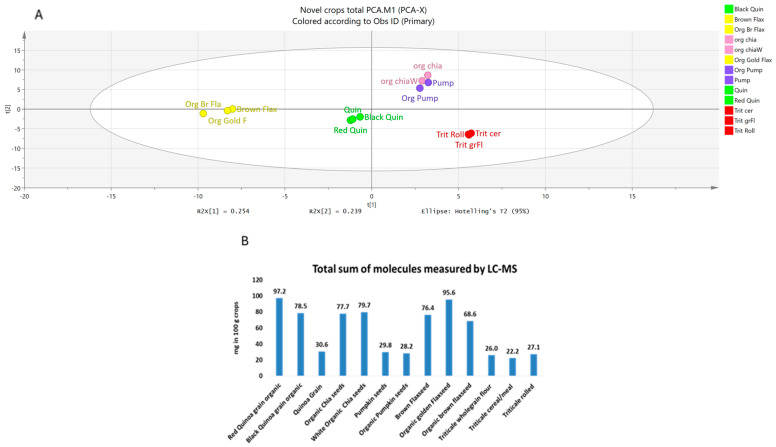

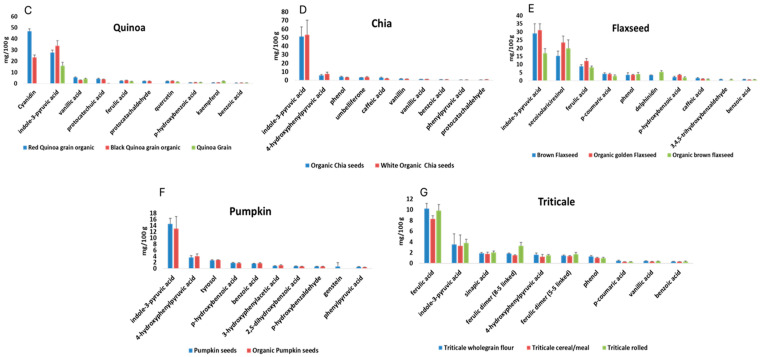
Principal component analysis (PCA), unit variance (UV) scaled plots for the first two PCs of all the plant metabolites that were detected in red quinoa grain organic, black quinoa grain organic, organic chia seeds, white organic chia seeds, pumpkin seeds, organic pumpkin seeds, brown flaxseed, organic golden flaxseed, organic brown flaxseed, triticale organic, triticale cereal/meal, triticale rolled (**A**). The sum of all molecules measured by LC-MS in each crop (summing the free, alkali-bound and acid-bound measured molecules) (**B**). Ten most abundant phytochemicals measured using targeted LC-MS/MS analysis and HPLC analysis (n = 3 ± STD) mg/kg in the crops (**C**–**G**). The rest of the phytochemicals measured in these crops are presented in Tables S6–S10 from Supplementary Materials.

Across all five crops, nutrient density and diversity were evident (Table 1; Figure 1, Figure 2 and Figure 3; Table 2 and Table 3). Pumpkin seeds emerged as the most protein-dense (≈29–36% by weight) and lipid-dense (≈42–45%), with values significantly greater than quinoa and triticale (*p* < 0.05). Chia and flaxseed provided the highest dietary fiber (≈11–15 g/100 g for flaxseed; ≈14–15 g/100 g for chia) and were characterized by exceptional omega-3 fatty acid (ALA) content (≈60% of total fat in chia; ≈52–60% in flaxseed). Quinoa and triticale contributed complex carbohydrates and distinctive phytochemical signatures, including anthocyanins in colored quinoa and ferulic acid in triticale. Collectively, these data provide an integrated compositional overview of selected commercially available products, highlighting complementary sources of protein, fiber, omega-3 fatty acids, and micronutrients. 

### 2.2. Protein Density and Amino Acid Quality

Pumpkin seeds contained the highest protein concentrations, followed by chia and flaxseed (Table 1). All crops contained the indispensable amino acids measured in this study (Table 2); however, tryptophan was not analyzed. Pumpkin seeds were particularly rich in leucine [16] and valine (≈1380–1395 mg/100 g), and chia exhibited the highest methionine among the crops measured. Based on UK RNI benchmarks (0.75 g/kg/day), a 100 g portion of pumpkin or chia can supply >50% of daily protein needs, especially for women (Table S1). While amino-acid content is an important component of protein quality, protein digestibility and bioavailability were not assessed in the present study. Plant proteins may present limiting amino acids, e.g., methionine/isoleucine in quinoa, leucine in pumpkin and flaxseed, threonine in chia; however, the nutritional implications of these patterns require further investigation incorporating digestibility measurements and protein-quality assessments. Yet these constraints can be overcome by complementary food combinations (e.g., cereals with legumes) and processing (fermentation, enzymatic treatments, heat) to reduce antinutrients and improve access to protein structures [17,18,19,20,21,22]. The observed amino-acid profiles may be relevant for food formulation and dietary planning, particularly where plant-based protein sources are used.

### 2.3. Lipid Content, Fatty Acid Profiles, and Cardiometabolic Relevance

Total fat differed significantly across crops (Table 1). Pumpkin seeds were lipid-dense and MUFA-rich (≈31–40% of total fat), dominated by oleic acid, whereas chia and flaxseed were PUFA-dominant, with ALA (n-3) at ≈60% (chia) and ≈52–60% (flaxseed) (Table 3). Quinoa contributed linoleic acid (n-6) (≈46–50%), with moderate MUFAs. These fatty-acid profiles are consistent with those previously reported for these crops in the literature. Higher ALA intake is associated with anti-inflammatory, lipid-lowering, and cardioprotective effects, and contributes to balancing Western dietary patterns often characterized by elevated n-6/n-3 ratios [15,23]. In our dataset, chia and flaxseed achieved low n-6/n-3 ratios (≈1:1), whereas pumpkin exhibited a higher n-6/n-3 ratio (≈30–33). These findings highlight substantial differences in fatty-acid composition among the analyzed products. At the RNI level, 100 g of chia or flaxseed can deliver ~56–93% of the PUFA recommendation, while pumpkin can meet a large share of MUFA targets (Tables S3 and S4). This data provides information on the relative contribution of the analyzed products to dietary fatty-acid intake. 

### 2.4. Dietary Fiber Quantity, NSP Diversity, and Gut Health

Dietary fiber was estimated from total NSP × 1.33, with chia and flaxseed providing the highest values (Table 1). Across crops, insoluble NSPs predominated, but chia also showed the highest soluble NSP (Figure 1). Monosaccharide profiling indicates pectic substances (uronic acids) in all crops; xyloglucans and arabinoxylans in chia/flaxseed; arabinogalactans and glucomannans in triticale, each previously associated with glycemic regulation, cholesterol metabolism, prebiotic activity, and immune modulation [24,25,26,27,28,29,30,31,32,33]. The diversity of fiber structures, rather than quantity alone, has been associated with microbial richness, SCFA production, vitamin biosynthesis, and intestinal barrier function. The analyzed products therefore provide diverse NSP architectures that may contribute to dietary fiber diversity [34]. 

### 2.5. Mineral Sufficiency and Public Health Implications

Mineral analysis (Figure 2) revealed high concentrations of essential micronutrients: magnesium (≈536–588 mg/100 g) and phosphorus (≈1069–1227 mg/100 g) in pumpkin; potassium (≈937–945 mg/100 g) in pumpkin/brown flaxseed; calcium (≈744 mg/100 g) and iron (≈9.1 mg/100 g) in white/organic chia; zinc in organic pumpkin (≈6.8 mg/100 g) and golden flaxseed (≈6.3 mg/100 g); selenium in triticale cereal/meal (≈46.5 µg/100 g); molybdenum in pumpkin (≈101–116 µg/100 g). At typical serving sizes, these profiles can meet or exceed RNIs for several minerals, e.g., magnesium and phosphorus with 100 g pumpkin; calcium and iron with 100 g chia based on total mineral content [10,35,36,37,38,39]. However, mineral bioavailability may be influenced by food-matrix components such as phytate, oxalate, fiber, and other antinutritional factors, which were not assessed in the present study. Importantly, these minerals can be delivered via natural fortification, incorporating pumpkin seed flour or chia/flax powders into baked goods, cereals, and plant-based beverages, while soaking, sprouting, fermentation, and phytase application have been reported to improve mineral bioaccessibility by reducing phytate and oxalate concentrations [40,41,42,43,44].

### 2.6. Phytochemical Richness and Functional Potential

Targeted LC-MS/MS and HPLC together screened for 171 phytochemicals across crops, with PCA resolving four distinct clusters: (i) chia and pumpkin (close clustering), (ii) flaxseed, (iii) quinoa, and (iv) triticale (Figure 3). This underscores crop-specific metabolite signatures and provides a framework for synergistic use or targeted selection in product design. Notable compounds included indole-3-pyruvic acid (dominant in chia; present in flaxseed, pumpkin, quinoa), a tryptophan metabolite which has been associated in previous studies with anti-inflammatory and neuroprotective activities; secoisolariciresinol and ferulic acid in flaxseed; cyanidin (anthocyanin) and quercetin in red/black quinoa; and 4-hydroxyphenylpyruvic acid in pumpkin [45,46,47,48,49]. These phytochemicals contribute to the distinct compositional profiles of the analyzed products and may be relevant for future investigations of their biological activity and technological applications. Technological advantages include natural colorants (e.g., cyanidin), antioxidants for shelf-life, and emulsifying properties, aligning with clean-label innovation and bioactive-enriched formulations [48]. RNI contributions are summarized in the text and detailed in Supplementary Tables S1–S5.

### 2.7. Summary Findings and Practical Implications

The integrated analysis demonstrates that quinoa, chia, flaxseed, pumpkin, and triticale possess complementary nutritional and phytochemical profiles: Protein and Amino Acids: High protein density (pumpkin; chia/flaxseed) with complementary indispensable amino acids, among those measured in the present study. Lipids: ALA-rich PUFA sources (chia/flaxseed) and pumpkin products rich in MUFAs (oleic acid) contribute distinct fatty-acid profiles. Fiber and NSPs: Diverse soluble/insoluble NSP architectures and monosaccharide compositions that may contribute to dietary fiber diversity. Minerals: Dense sources of magnesium, phosphorus, potassium, calcium, iron, zinc, selenium, and molybdenum based on total mineral content. Phytochemicals: Distinct metabolite clusters and diverse phytochemical profiles, including indoles, lignans, phenolic acids, anthocyanins, and flavonols, which may be of interest for future food and nutrition research. 

Taken together, strategic integration of these crops in mainstream diets and product formulations has the potential to increase dietary diversity with respect to protein, fatty acids, fiber, minerals, and phytochemicals. The present findings provide detailed compositional information on selected commercially available products and may support future food formulation, dietary assessment, and nutrition research initiatives. This unified perspective moves beyond single-nutrient evaluations to highlight the value of examining multiple nutritional and phytochemical components simultaneously [3,4,11].

### 2.8. Study Limitations

Although this study provides an extensive compositional analysis of five UK-available plant-based crops, certain limitations must be acknowledged. First, the research focused on raw, commercially sourced samples, representing selected products available on the UK market, which may not fully represent variability because of geographic origin, soil composition, seasonal factors, cultivar differences, and post-harvest handling. Furthermore, the analyzed samples should not be considered representative of all commercially available products within each crop category. Second, while nutrient and phytochemical concentrations were quantified, bio-accessibility/bioavailability and digestibility were not assessed; antinutritional factors such as phytates and oxalates could influence absorption and utilization. Consequently, the reported values reflect total nutrient concentrations rather than bioaccessible or bioavailable fractions. Third, the study employed targeted LC-MS/MS and HPLC screening of 171 compounds, which, although thorough, does not capture the entire metabolome or potential synergistic interactions among bioactive compounds. Finally, the absence of clinical or *in vivo* validation limits direct translation of compositional data into health outcomes, necessitating caution when extrapolating functional benefits. In addition, no environmental, life-cycle, or sustainability assessments were conducted; therefore, conclusions regarding sustainability should be interpreted within the broader context of the existing literature rather than as direct outcomes of the present study.

### 2.9. Suggestions for Future Research

Future investigations should prioritize bioavailability studies using simulated digestion models and human intervention trials to establish the physiological relevance of these compositional findings. Expanding metabolomic coverage through untargeted LC-MS/MS and integrating proteomic and lipidomic approaches could uncover additional bioactive compounds and metabolic pathways. Research on processing techniques, such as sprouting, fermentation, and extrusion, should explore their impact on nutrient retention, phytochemical enhancement, and reduction in antinutritional factors. Furthermore, combining compositional analysis with environmental sustainability metrics, such as life-cycle assessment and carbon footprint analysis, would strengthen understanding of the relationships between nutritional composition and environmental sustainability. Finally, exploring the effects of individual crops and crop combinations on gut microbiota composition, nutrient bioavailability, and relevant physiological biomarkers could inform the development of optimized plant-based dietary patterns and functional food formulations.

### 2.10. Practical Applications

The findings of this study provide detailed compositional information that may be relevant to public health nutrition, food product development, and future research on plant-based foods. Incorporating nutrient-dense crops such as pumpkin, chia, flaxseed, quinoa, and triticale into mainstream diets may contribute to greater dietary diversity with respect to protein, fiber, minerals, fatty acids, and phytochemicals. The distinct nutritional and phytochemical profiles identified across the analyzed products may be of interest for the development of plant-based and clean-label food products. In addition, the compositional differences observed among crops highlight opportunities for their use in food formulation and dietary diversification strategies. As no measurements of bioavailability, physiological responses, health outcomes, or environmental sustainability were undertaken, the present findings should be interpreted as compositional evidence that can inform future nutritional, technological, and sustainability-focused investigations. 

## 3. Materials and Methods

### 3.1. Standards and Reagents

The standards and general laboratory reagents were purchased from Sigma-Aldrich (Gillingham, UK) and Fisher Scientific UK Ltd. (Loughborough, UK), each of high purity (>95%) or synthesized as described previously [49,50]. Chemicals used for ICP-MS analysis were nitric acid of TraceSelect Ultra grade (Fluka, Sigma-Aldrich, Buchs, Switzerland), hydrochloric acid (30%) of Ultrapur grade (Merck, Darmstadt, Germany), and deionized water (Millipore, Bedford, MA, USA). Single element standards were purchased from Inorganic Ventures (Christiansburg, VA, USA).

### 3.2. Materials

Red quinoa grain organic (Bolivia), black quinoa grain organic (Peru), quinoa grain (Peru), organic chia seeds (Argentina), white organic chia seeds (Argentina), pumpkin seeds (Poland), organic pumpkin seeds (Poland), brown flaxseed (Belgium), organic golden flaxseed (Kazakhstan), organic brown flaxseed (China), were purchased from Buywholefoodsonline.co.uk (an online UK supermarket), and triticale whole grain flour, triticale Cereal/meal, triticale rolled purchased online from Bob’s red Mill (Canada and the northern U.S.). Products were purchased in the UK during the study period (2013–2014) and analyzed as commercially available retail products. Product information, including country of origin and batch/lot numbers, was recorded where available from product packaging and supplier documentation. All seed and grain samples were freeze-milled (Spex 6700; Edison, NJ, USA) and stored under vacuum in a desiccator prior to analysis. Analytical technical (n = 3) replicates were performed on homogenized milled material obtained from each product sample. All the data is reported per material as-purchased basis.

### 3.3. Macronutrient Analysis

The macronutrient content of seeds, grains and flours was analyzed using routine proximate analysis as described before [51]. 

#### 3.3.1. Analysis of Protein

The protein concentration was analyzed based on the Dumas combustion method previously published [52]. Protein was determined by measuring crude nitrogen using a Vario Max CN analyzer (Elementar; Stockport, UK) and then multiplying the result by 6.25. Protein content was calculated using a nitrogen-to-protein conversion factor of 6.25, which assumes an average nitrogen content of 16% in proteins. 

#### 3.3.2. Analysis of Total Fat and Fatty Acids

The total fat analysis was done based on a method published previously [53]. Briefly, lipid extraction was carried out using a chloroform solvent system. Following phase separation, the organic layer was collected, filtered, and evaporated to dryness under reduced pressure. Total fat content was determined gravimetrically from the recovered lipid fraction. All analyses were performed in triplicate (n = 3). For the analysis of the fatty acid methyl esters (FAMEs), the fat was first extracted and analyzed by GC. Samples (in the range of 50–200 mg) were suspended in methanol (2 mL) and macerated using an Ultra Turrax homogenizer (IKA T10, Staufen, Germany) at setting 4 (2800× *g*; 30 s at 5 °C). Then, chloroform (4 mL) was added, and the samples macerated again for a further 2 min and the supernatant separated and retained. The remaining precipitates were extracted with chloroform/methanol (5 mL, 2:1) and the supernatant was isolated and combined with the first supernatant obtained. Deionized water (6 mL) was added, and the samples were cen-trifuged at 1800× *g* (5 min, 5 °C) to separate the lower chloroform layer. Then, a gentle stream of nitrogen was further used to remove chloroform. An aliquot of the extracted fat (20 mg) was mixed with hexane (0.5 mL) and methanolic HCl (methanol, 20 mL; acetyl chloride, 2 mL). Then, the samples were suspended in an internal standard of heptadecanoic acid (0.01 mL, 2 mg/mL in methanol). The samples were heated in a block heater at 105 °C for 2 h and, subsequently, taken out to allow them to cool down. Then, distilled water (2.5 mL) and hexane (2.5 mL) were added to the samples, and the top layers were separated by centrifugation at 1800× *g* (5 min, 5 °C). The samples were then extracted twice more with 2.5 mL of diethyl ether. The layers obtained were combined and mixed with distilled water (4 mL), and the lower aqueous layers produced were aspirated off. A volume of 5 mL distilled water was added to the samples, and the top layers were placed into stoppered tubes containing anhydrous sodium sulphate (1–2 cm). The samples were shaken well to separate any water from the solvents, and the solvents were evaporated under a stream of nitrogen (40 °C). The residues were mixed with butylated hydroxytoluene (0.1 mL; 0.02% in hexane) and then transferred to GC vials.

The separation of the compounds was performed by gas chromatography (Agilent Technologies 6890N, Stockport, UK) equipped with a CP-SIL 88, 50 m × 0.25 mm column. The carrier gas was helium using a flow of approximately 0.8 mL/minute at a pressure of 16 psi. The temperatures of the injector and detector were 250 °C and 270 °C, respectively. The oven temperature, registering 80 °C initially, was raised to 160 °C for 3 min, then further increased to 190 °C, with a final temperature of 230 °C that was maintained for 18 min.

#### 3.3.3. Analysis of Moisture Content

The moisture content was determined according to an AOAC method [54]. Samples (2.5–5.0 g; n = 3) were dried in an oven at 100 °C to constant weight, cooled in a desiccator, and reweighed. Moisture content was calculated from the difference between initial and final sample weights. 

#### 3.3.4. Analysis of Ash

Ash content was determined according to a method published previously [54]. Following moisture determination, samples (n = 3) were incinerated in a muffle furnace at 550 °C for 3 h. The resulting ash was cooled in a desiccator and weighed to determine total ash content. 

#### 3.3.5. Analysis of Soluble and Insoluble Non-Starch Polysaccharides

The soluble and insoluble non-starch polysaccharide content was determined using previously published methods [55]. The reagents and standards used included allose (internal standard), sodium acetate buffer (pH 5.2), sodium chloride/boric acid solution, sodium borohydride, α-amylase, and a combined pullulanase–pancreatic enzyme preparation for starch hydrolysis. 

Sample preparation: Samples containing more than 5% fat were defatted prior to NSP analysis using acetone extraction. Defatted samples (0.3 g, n = 3) were subsequently subjected to aqueous extraction to isolate soluble NSP fractions, while insoluble NSP fractions were recovered from the remaining residue. Soluble and insoluble fractions were treated separately throughout the analytical procedure.

To remove starch, both soluble and insoluble NSP fractions underwent enzymatic hydrolysis using α-amylase, pullulanase, and pancreatic enzymes under controlled temperature conditions. Following enzymatic treatment, NSPs were precipitated with ethanol, washed with ethanol and acetone, and recovered by centrifugation. The isolated NSP fractions were hydrolyzed with sulfuric acid to release constituent monosaccharides. Neutral sugars were subsequently converted to alditol acetates through reduction and derivatization procedures and analyzed by gas chromatography (Hewlett Packard HP7890N, Palo Alto, CA, USA) equipped with an SP2330 column. Uronic acids were determined colorimetrically using the 3,5-dimethylphenol method and quantified spectrophotometrically. Monosaccharide composition data were used to calculate soluble and insoluble NSP contents and to characterize NSP structural composition. All analyses were performed in triplicate (n = 3). GC analysis of sugars: The separation of sugars was performed by gas chromatography using a Hewlett Packard HP7890N system equipped with an SP2330 capillary column (30 m × 0.75 mm). Chromatographic separation was achieved using a temperature programme ranging from 180 °C to 220 °C, with helium as the carrier gas. The injector and detector temperatures were maintained at 250 °C and 275 °C, respectively. Samples were injected at a volume of 1 μL. 

### 3.4. Micronutrient Analysis

A previously published method by Multari et al. [56] was used to determine mineral concentrations. Briefly, samples (0.4 g, n = 3) were digested with nitric acid (8 mL, 65% *v*/*v*) and deionized water (1 mL) using microwave-assisted digestion (MARS 6, CEM; Matthews, NC, USA). Digestion was performed using a two-stage temperature programme, increasing from 20 °C to 150 °C over 15 min, followed by heating from 150 °C to 165 °C over 10 min and maintaining this temperature for 20 min.

The isotopes analyzed by ICP-MS included ^23Na, ^24Mg, ^31P, ^39K, ^44Ca, ^51V, ^52Cr, ^55Mn, ^56Fe, ^59Co, ^60Ni, ^63Cu, ^66Zn, ^78Se, ^95Mo, ^111Cd, ^202Hg, and ^208Pb. All standards and samples were prepared in a matrix containing nitric acid (2% *v*/*v*) and hydrochloric acid (0.5% *v*/*v*).

Mineral concentrations were determined using an Agilent 7700X ICP-MS spectrometer (Agilent Technologies UK; Cheadle, UK) equipped with a MicroMist nebulizer and nickel sampler and skimmer cones. Erbium (1 mg/L) was used as the internal standard. Instrument operation and data acquisition were performed according to the procedure described by Multari et al. [56].

### 3.5. Amino Acid Analysis

Apart from tryptophan, which was not analyzed in this study (due to analytical methodology limitations), amino acid analysis was performed according to the AOAC Official Method 985.28 [57]. Acid hydrolysis was used for the determination of histidine (His), serine (Ser), arginine (Arg), glycine (Gly), aspartic acid (Asp), glutamic acid (Glu), threonine (Thr), alanine (Ala), proline (Pro), lysine (Lys), tyrosine (Tyr), valine (Val), isoleucine (Ile), leucine (Leu), and phenylalanine (Phe), while performic acid oxidation followed by hydrolysis was used for methionine (Met) and cysteine (Cys). Amino acids were quantified by ultra-performance liquid chromatography (UPLC) following AccQ-Tag derivatization, as previously described [51,57].

The AccQ-Tag method is a pre-column derivatization technique that enables the separation and quantification of amino acids by reversed-phase UPLC with ultraviolet detection. Analyses were performed using an Acquity UPLC system equipped with an AccQ-Tag Ultra C18 column (1.7 μm, 2.1 × 100 mm), quaternary solvent manager, column oven, and tunable UV detector set at 260 nm. Chromatographic separation was performed using the solvent system and gradient conditions specified in the Waters AccQ-Tag application protocol and described previously [51,57].

### 3.6. Phytochemicals Analysis

The seeds, grains, and flours were extracted for the quantification of simple phenols, benzoic acids, phenolic acids, phenylacetic acids, phenylpropionic acids, phenylpyruvic acids, phenyllactic acids, mandellic acids, phenolic dimers, acetophenones, benzaldehydes, cinnamaldehydes, benzyl alcohols, cinnamyl alcohols, indoles, isoflavones, coumarins, chalcones, flavanones, flavones, flavonols, and anthocyanidins.

The method used for the extraction of phenolic compounds (excluding anthocyanidin aglycones) was based on previously published procedures [51,58]. Briefly, samples (approximately 0.1 g dry weight; n = 3) were subjected to sequential extraction to obtain free and bound phenolic fractions. Free phenolics were extracted into ethyl acetate under acidic conditions, while bound phenolics were released by sequential alkaline and acid hydrolysis followed by ethyl acetate extraction. The resulting extracts were evaporated to dryness and stored at −70 °C prior to LC–MS/MS analysis.

Anthocyanidins were extracted and hydrolyzed using a method adapted from Zhang et al. [46]. Briefly, samples (approximately 0.1 g dry weight; n = 3) were extracted with methanol:water:37% HCl) (50:33:17, *v*:*v*:*v*), and the combined extracts were hydrolyzed at elevated temperature to release anthocyanidin aglycones. Following cooling, samples were analyzed by HPLC.

Phytochemical identification and quantification were performed using targeted LC–MS/MS and HPLC methods as previously described [46,51,58].

#### 3.6.1. Preparation of the Extracts for LC–MS/MS Analysis

Aliquots (0.1 mL) of the extracts were mixed with internal standard 1 (IS1; ^13C-benzoic acid) for negative-ion mode analysis and internal standard 2 (IS2; 2-amino-3,4,7,8-tetramethylimidazo[4,5-f]quinoxaline) for positive-ion mode analysis prior to LC–MS/MS quantification. 

##### LC–MS/MS Analysis

For the LC–MS/MS analysis previously published methods have been used [51,58,59,60]. The plant metabolites were separated on an Agilent 1100 HPLC system (Agilent Technologies; Wokingham, UK) fitted with a Zorbax Eclipse 5 μm, 150 mm × 4 mm column (Agilent Technologies) according to a previously published method [58]. This included mainly benzoic acids, benzaldehydes, benzenes, acetophenones, cinnamic acids, phenylpropionic, phenylacetic, phenylpyruvic, phenyllactic acids, flavonoids, isoflavonoids, catechins and lignans. The eluents acquired during the liquid chromatography process were directly transferred to an ABI 3200 triple quadrupole mass spectrometer (Applied Biosystems; Warrington, UK) equipped with a Turbo Ion Spray™ (TIS) source. The gradients consisted of water with 0.1% acetic acid and acetonitrile with 0.1% acetic acid. Method 1: 40–90% B (13 min), 90% B (1 min), 90–40% B (1 min), 40% B (9 min); method 2: 10–55% B (45 min), 55–80% B (15 min), 80% B (3 min), 80–10% B (0.2 min), 10% B (4.8 min) and method 3: 50–80% B (10 min), 80% B (2 min), 80–50% B (1 min), 50% B (4 min). The volume used for the injection of the samples was 5 μL with a flow rate of 300 μL/minute. The compounds in the samples underwent evaluation through multiple reaction monitoring, while the standard solutions were put into the system using a syringe pump. The ion transitions in each of the metabolites were identified according to their molecular ions and fragment ions. The elution time was employed to identify analytes containing the same type of molecular and fragment ions. For each analyte the voltage parameters were optimized one by one. This included declustering potential, collision energy and cell entrance/exit potentials. For all the phytochemical quantifications, external standard calibrations curves were prepared in concentration intervals of 2 ng μL^−1^ up to 10 pg μL^−1^. The threshold used for quantification had a signal-to-noise ratio of 3 to 1. All the ion transitions for each of the metabolites were determined based upon their molecular ions and strong fragment ions; their voltage parameters; declustering potential, collision energy and cell entrance/exit potentials were optimized individually for each metabolite and have been previously described [51,58,59,60].

##### HPLC Analysis of Anthocyanidins

Quantification of anthocyanidins was performed as previously described [51] using an Agilent 1260 Infinity HPLC system (Agilent Technologies; Wokingham, UK) equipped with a quaternary pump, diode-array detector (DAD), thermostatted autosampler, and column oven. Separation was achieved using a Synergi Polar-RP column (4 μm, 250 × 4.6 mm) fitted with a Polar-RP guard column (Phenomenex; Macclesfield, UK).

DAD spectra were recorded between 200 and 700 nm, and chromatograms were monitored at 530 nm. The mobile phases consisted of (A) formic acid (2.125%) and (B) acetonitrile (85:15, *v*/*v*). Chromatographic separation was performed using the method adapted from Zhang et al. [46], employing an isocratic elution of 18% B at a flow rate of 1 mL min^−1^ and a column temperature of 35 °C.

Anthocyanidins were identified and quantified using external calibration with delphinidin, cyanidin, petunidin, pelargonidin, peonidin, and malvidin standards. Limits of detection (LOD) and limits of quantification (LOQ) for the individual standards were determined as previously reported [51].

### 3.7. Statistical Analysis

All nutrient and phytochemical data were obtained from three technical replicates and are presented as mean ± standard deviation. 

Phytochemical data were analyzed by principal component analysis (PCA) following unit variance (UV) scaling using SIMCA version 14.1 (Umetrics, Cambridge, UK).

## 4. Conclusions

This study provides a comprehensive compositional characterization of commercially available quinoa, chia, flaxseed, pumpkin seed, and triticale products available in the UK market. Pumpkin seeds provide the highest protein (up to approximately 36%) and fat (approximately 45%) content, making them a valuable plant-based source of protein and lipids, while chia and flaxseed deliver exceptional dietary fiber (11–18 g/100 g) and omega-3 fatty acids, promoting gut and cardiometabolic health. Mineral analysis revealed high concentrations of calcium, iron and manganese in chia products and high concentrations of phosphorus, magnesium, and potassium in pumpkin seed products. Targeted LC-MS/MS profiling identified red quinoa, golden flaxseed, and white chia as phytochemically dense (up to 97.2 mg/100 g), highlighting substantial variation in phytochemical composition among the analyzed products. These findings highlight the complementary nutritional and phytochemical characteristics of the analyzed crops and their potential contribution to dietary nutrient and phytochemical diversity. The results may support future food formulation, dietary assessment, and nutrition research initiatives involving plant-based foods. Further studies are required to assess nutrient bioavailability, digestibility, physiological effects, and environmental sustainability metrics before conclusions can be drawn regarding health outcomes or sustainability impacts. 

## Figures and Tables

**Table 1:** Macronutrient content in g per 100 g (n = 3 ± STD) milled crops: protein, total fat, total carbohydrate, resistant starch, total (NSP) non-starch polysaccharides, including (ash, dry matter) for red quinoa grain organic, black quinoa grain organic, organic chia seeds, white organic chia seeds, pumpkin seeds, organic pumpkin seeds, brown flaxseed, organic golden flaxseed, organic brown flaxseed, triticale organic, triticale cereal/meal, triticale rolled.

Samples	Dry Matter	Ash	Total Fat (%RNI Contribution) ^1^	Total Carbohydrates	Resistant Starch	Protein (%RNI Contribution) ^2^	Fibre ^3^ (%RNI Contribution) ^4^
**Quinoa (*Chenopodium* *Quinoa*)**							
Red grain organic	91.7 ± 0.05	2.32 ± 0.09	5.52 ± 0.28 (6.39)	86.80 ± 3.29	0.34 ± 0.02	12.65 ± 0.16 (25.34)	7.32 ± 0.15 (24.41)
Black grain organic	91.54 ± 0.08	2.50 ± 0.06	5.82 ± 0.21 (6.73)	76.01 ± 2.43	0.48 ± 0.02	14.25 ± 0.23 (28.56)	8.53 ± 0.39 (28.45)
Grain	91.75 ± 0.09	2.08 ± 0.11	5.89 ± 0.14 (6.81)	88.08 ± 0.97	0.66 ± 0.03	12.54 ± 0.44 (25.13)	6.35 ± 1.42 (21.18)
**Chia seeds (*Salvia hispanica*)**							
Organic seeds	94.39 ± 0.05	4.84 ± 0.12	27.64 ± 0.37 (31.96)	0.86 ± 0.45	0.00 ± 0.00	25.58 ± 0.89 (51.27)	14.88 ± 0.59 (49.59)
White Organic seeds	95.68 ± 0.07	5.14 ± 0.12	31.06 ± 0.50 (35.92)	1.24 ± 0.22	0.00 ± 0.00	24.04 ± 1.47 (48.18)	15.16 ± 0.71 (50.52)
**Pumpkin seeds (*Cucurbita pepo*)**							
Seeds	96.26 ± 0.06	4.24 ± 0.04	45.06 ± 0.08 (52.11)	2.65 ± 0.46	0.00 ± 0.00	28.75 ± 6.09 (57.61)	7.00 ± 0.16 (23.34)
Organic seeds	95.26 ± 0.51	4.83 ± 0.04	41.68 ± 0.67 (48.20)	4.48 ± 1.02	0.00 ± 0.00	35.83 ± 0.74 (71.81)	7.18 ± 0.10 (23.92)
**Flaxseed (*Linum usitatissimum*)**							
Brown	95.41 ± 0.89	3.30 ± 0.06	37.96 ± 0.10 (43.90)	3.79 ± 0.59	0.00 ± 0.00	23.52 ± 0.07 (47.14)	14.99 ± 1.02 (49.96)
Organic golden	95.38 ± 0.13	3.07 ± 0.03	35.28 ± 0.67 (40.80)	3.68 ± 0.00	0.00 ± 0.00	25.69 ± 0.22 (51.48)	12.18 ± 0.26 (40.59)
Organic brown	95.98 ± 0.16	3.04 ± 0.05	38.76 ± 0.63 (44.82)	5.11 ± 0.59	0.05 ± 0.08	22.00 ± 0.11 (44.09)	11.30 ± 0.37 (37.68)
**Triticale (*Triticosecale*)**							
Whole grain flour	90.64 ± 0.98	1.79 ± 0.04	2.81 ± 0.35 (3.25)	74.71 ± 2.07	0.17 ± 0.04	13.15 ± 0.14 (26.34)	10.61 ± 0.30 (35.36)
Cereal/meal	91.05 ± 0.08	1.78 ± 0.02	2.19 ± 0.32 (2.53)	79.31 ± 8.41	0.22 ± 0.00	12.92 ± 0.37 (25.88)	9.38 ± 0.56 (31.27)
Rolled	91.86 ± 0.04	1.70 ± 0.04	1.83 ± 0.12 (2.12)	76.92 ± 2.22	0.20 ± 0.01	14.10 ± 0.07 (28.26)	8.92 ± 0.04 (29.74)

RNI is Reference Nutrient Intake [13]. (^1^) represents % contribution to RNI for Fat (97 g/day for males, 78 g/day for females) [13] and this has been calculated as average. (^2^) represents % contribution to RNI for Protein (55.5 gr/day for males, 45 g/day for females) [13] and this has been calculated as average. (^3^) value for dietary fiber, calculated as Fiber = 1.33x total NSP. (^4^) represents % contribution to RNI for fiber (30 g/day) [14].

**Table 2:** Essential and non-essential amino acids content (excepting tryptophan) in mg per 100 g (n = 3 ± STD) milled crops for red quinoa grain organic, black quinoa grain organic, organic chia seeds, white organic chia seeds, pumpkin seeds, organic pumpkin seeds, brown flaxseed, organic golden flaxseed, organic brown flaxseed, triticale organic, triticale cereal/meal, triticale rolled.

Essential Amino Acids									
Samples	His	Ileu	Leu	Lys		Met	Phe(+ Tyr)	Thr	Val
**Quinoa *Chenopodium Quinoa*)**									
Red grain organic	273.60 ± 11.59 ^¥^	364.21 ± 27.42 ^¥^	633.53 ± 47.10 ^¥^	569.61 ± 54.08 ^¥^		243.37 ± 3.79^↙^	374.02 ± 21.89 ^↙^	362.41 ± 22.83 ^¥^	459.18 ± 30.67 ^¥^
Black grain organic	271.89 ± 25.87 ^¥^	363.40 ± 27.71 ^¥^	627.19 ± 50.13 ^¥^	572.19 ± 38.03 ^¥^		566.26 ± 11.03 ^¥^	375.80 ± 31.02 ^↙^	368.23 ± 29.52 ^¥^	466.71 ± 34.16 ^¥^
Grain	228.12 ± 19.17 ^↙^	307.71 ± 10.09 ↙	527.58 ± 20.35 ^¥^	479.46 ± 7.18 ^¥^		288.39 ± 40.68 ^↙^	311.21 ± 23.23 ^↙^	311.04 ± 15.36 ^¥^	397.34 ± 11.34 ^¥^
**Chia seeds (*Salvia hispanica*)**									
Organic seeds	503.50 ± 77.79 ^§^	700.98 ± 133.14 ^§^	1312.28 ± 249.06 ^§^	935.32 ± 171.51 ^§^		252.28 ± 3.63 ^↙^	970.26 ± 165.09 ^§^	721.80 ± 116.07 ^§^	940.83 ± 172.18 ^§^
White Organic seeds	488.93 ± 84.41 ^¥^	419.99 ± 364.93 ^¥^	777.02 ± 676.94 ^§^	594.72 ± 518.55 ^¥^		939.73 ± 97.96 ^§^	556.93 ± 484.19 ^¥^	430.31 ± 373.73 ^§^	572.59 ± 497.31 ^§^
**Pumpkin seeds (*Cucurbita pepo*)**									
Seeds	599.21 ± 15.67 ^§^	1053.95 ± 61.52 ^§^	1991.61 ± 110.70 ^§^	1051.72 ± 92.65 ^§^		715.77 ± 30.19 ^§^	1364.64 ± 25.27 ^§^	823.98 ± 43.95 ^§^	1394.82 ± 91.33 ^§^
Organic seeds	604.30 ± 38.91 ^§^	1038.80 ± 55.18 ^§^	2016.67 ± 121.30 ^§^	1138.39 ± 48.32 ^§^		795.29 ± 4.91 ^§^	1340.59 ± 80.45 ^§^	848.85 ± 45.61 ^§^	1379.59 ± 65.36 ^§^
**Flaxseed (*Linum usitatissimum*)**									
Brown	349.98 ± 25.12 ^↙^	716.22 ± 67.75 ^§^	1035.48 ± 97.78 ^§^	701.03 ± 91.41 ^§^		410.21 ± 152.49 ^↙^	787.34 ± 51.46 ^§^	670.96 ± 56.15 ^§^	899.06 ± 79.73 ^§^
Organic golden	376.33 ± 3.08 ^¥^	804.86 ± 29.79 ^§^	1129.82 ± 32.05 ^§^	774.76 ± 27.33 ^§^		247.19 ± 7.35 ^↙^	851.32 ± 19.11 ^§^	715.16 ± 13.72 ^§^	995.56 ± 27.92 ^§^
Organic brown	329.00 ± 14.58 ^¥^	676.32 ± 33.91 ^§^	992.92 ± 43.13 ^§^	682.08 ± 28.52 ^§^		423.01 ± 18.59 ^↙^	720.43 ± 36.03 ^¥^	642.80 ± 27.35 ^§^	863.89 ± 36.12 ^§^
**Triticale (*Triticosecale*)**									
Whole grain flour	227.44 ± 3.80 ^↙^	344.92 ± 15.10 ^¥^	684.87 ± 32.41 ^¥^	338.18 ± 29.46 ^↙^		224.63 ± 22.67 ^↙^	475.87 ± 6.80 ^¥^	336.43 ± 17.42 ^¥^	481.06 ± 22.28 ^¥^
Cereal/meal	233.93 ± 17.24 ^↙^	366.46 ± 25.75 ^¥^	711.97 ± 49.57 ^¥^	352.46 ± 45.50 ^↙^		104.29 ± 3.35 ^↙^	484.51 ± 24.09 ^¥^	338.79 ± 29.82 ^¥^	507.90 ± 43.75 ^¥^
Rolled	179.36 ± 8.97 ^↙^	435.22 ± 1.14 ^¥^	842.90 ± 6.43 ^§^	430.68 ± 15.61 ^¥^		222.63 ± 11.58 ^↙^	538.85 ± 9.05 ^¥^	378.72 ± 7.15 ^¥^	593.32 ± 3.76 ^§^
**Non-Essential Amino Acids**									
**Samples**	**Tyr**	**Ala**	**Asp**	**Glu**	**Gly**	**Pro**	**Cys**	**Ser**	**Arg**
**Quinoa (*Chenopodium Quinoa*)**									
Red grain organic	274.44 ± 8.19	429.57 ± 32.99	2180.27 ± 150.97	1427.80 ± 81.36	5011.78 ± 272.89	371.89 ± 24.04	223.22 ± 6.84	443.47 ± 22.49	870.99 ± 20.14
Black grain Organic	270.74 ± 31.10	435.60 ± 30.63	2157.41 ± 142.76	1429.92 ± 100.93	5382.71 ± 416.77	376.34 ± 29.14	445.19 ± 11.28	441.68 ± 38.08	851.93 ± 82.57
Grain	250.95 ± 19.67	359.84 ± 9.25	1780.73 ± 42.22	1165.15 ± 37.89	4382.28 ± 249.84	311.69 ± 12.78	264.67 ± 27.78	370.67 ± 20.87	723.65 ± 52.56
**Chia seeds (*Salvia hispanica*)**									
Organic seeds	617.27 ± 109.63	984.39 ± 173.66	4730.69 ± 807.21	3552.39 ± 639.51	9293.76 ± 1213.18	750.22 ± 125.92	222.10 ± 3.47	1112.91 ± 191.58	2090.50 ± 352.42
White Organic seeds	369.48 ± 321.07	584.11 ± 507.58	2712.53 ± 2349.94	2118.71 ± 1840.44	5634.18 ± 4878.84	454.12 ± 394.61	651.90 ± 63.69	659.55 ± 571.91	1247.92 ± 1083.59
**Pumpkin seeds (*Cucurbita pepo*)**									
Seeds	841.06 ± 22.58	1222.19 ± 85.23	6215.95 ± 480.36	5010.35 ± 391.74	14,829.59 ± 441.30	1019.48 ± 46.54	277.96 ± 11.60	1426.66 ± 72.15	4228.30 ± 160.44
Organic seeds	905.10 ± 79.14	1285.15 ± 59.02	6655.60 ± 289.15	5342.62 ± 231.73	15,482.35 ± 949.27	1044.34 ± 60.50	549.20 ± 10.96	1476.69 ± 91.60	4320.33 ± 281.32
**Flaxseed (*Linum usitatissimum*)**									
Brown	422.92 ± 32.80	817.17 ± 84.85	4260.90 ± 437.93	3516.99 ± 390.30	10,355.23 ± 717.39	663.90 ± 57.28	559.02 ± 205.63	863.85 ± 69.42	1728.85 ± 132.00
Organic golden	448.06 ± 10.45	887.38 ± 29.82	4714.78 ± 185.08	3919.35 ± 160.46	10,854.04 ± 143.47	714.93 ± 17.66	319.96 ± 7.63	914.86 ± 22.88	1894.63 ± 34.89
Organic brown	395.80 ± 14.19	770.42 ± 29.58	4044.74 ± 157.2	3348.74 ± 129.28	10,002.25 ± 368.28	630.92 ± 22.18	574.13 ± 29.13	820.69 ± 31.15	1581.67 ± 68.0
**Triticale (*Triticosecale*)**									
Whole grain flour	295.46 ± 17.34	406.55 ± 26.35	1629.40 ± 174.59	2844.67 ± 157.69	4579.45 ± 145.30	1045.19 ± 40.85	322.98 ± 17.73	511.20 ± 24.25	533.92 ± 31.04
Cereal/meal	251.76 ± 21.35	414.37 ± 44.97	1596.05 ± 155.90	3054.08 ± 233.01	4613.33 ± 397.45	1091.46 ± 71.90	146.12 ± 3.93	518.04 ± 34.21	507.51 ± 52.74
Rolled	305.42 ± 16.92	486.73 ± 10.65	1887.64 ± 36.38	3695.59 ± 153.04	5033.44 ± 105.90	1293.71 ± 51.37	323.96 ± 13.31	1162.96 ± 22.76	606.45 ± 15.38

(^↙^) represents the contribution of <50%; (^¥^) represents the contribution of 50–79%; (^§^) >80% of protein requirements (g/kg per day) for amino acid requirements (mg/kg per day), contribution, men 75 kg, women 60 kg, % females, % males, contribution calculated as average. (His), histidine 8 mg/kg/d; (Ile), isoleucine 10 mg/kg/d; (Leu), leucine 14 mg/kg/d; (Lys), lysine 12 mg/kg/d; (Thr), Threonine 7 mg/kg/d; (met), methionine 10 mg/kg/day; (Phe), phenylalanine + Tyrosine, (Tyr) 14 mg/kg/day; (Val), valine 10 mg/kg/day [15]; Trp (nd).

**Table 3:** Composition of fatty acids as % of total fat for red quinoa grain organic, black quinoa grain organic, organic chia seeds, white organic chia seeds, pumpkin seeds, organic pumpkin seeds, brown flaxseed, organic golden flaxseed, organic brown flaxseed, triticale organic, triticale cereal/meal, triticale rolled.

Samples		Saturated Fatty Acids			Total Saturated Fatty Acid (SFA)	Monounsaturated Fatty		Acids (MUFA)	Ratio Omega-6/Omega-3	Polyunsaturated Fatty Acids (PUFA)				Total PUFA (RNI Contribution%) ^5^
		Hexadecanoic Acid (Palmitic Acid)	Octadecanoic Acid (Stearic Acid)	Tetracosanoic Acid (Lignoceric Acid)		Cis-9-Octadecenoic Acid ^1^	Cis-11-Octadecenoic Acid	Total MUFA (RNI Contribution%) ^4^		Cis,Cis-9,12-Octadecadienoic Acid (Linoleic Acid) ^2^	Cis,Cis,Cis-9,12,15-Octadecatrienoic Acid (α-Linolenic Acid) ^3^	Trans,Trans-9,11-Octadecadienoic ACID	Cis,Cis,Cis-11,14,17-Eicosatrienoic Acid	
Quinoa (*Chenopodium Quinoa*)	Red grain organic	9.75 ± 0.05	1.02 ± 0.08	0.48 ± 0.00	12.38	30.30 ± 0.04	0.00 ± 0.00	30.67 (10.75)	4.69	45.77 ± 0.09	8.43 ± 0.09	0.00 ± 0.00	1.38 ± 0.01	56.6 (9.73)
	Black grain organic	10.77 ± 0.17	1.18 ± 0.11	0.33 ± 0.00	13.64	26.31 ± 0.17	0.00 ± 0.00	26.79 (9.90)	2.79	50.29 ± 0.30	6.37 ± 0.15	0.01 ± 0.02	1.49 ± 0.00	59.06 (10.70)
	Grain	9.75 ± 0.05	1.02 ± 0.08	0.48 ± 0.00	12.3	30.30 ± 0.04	0.00 ± 0.00	30.67 (11.47)	2.22	45.77 ± 0.09	8.43 ± 0.09	0.00 ± 0.00	1.38 ± 0.00	56.6 (10.38)
Chia seeds (*Salvia hispanica*)	Organic seeds	7.55 ± 0.11	3.35 ± 0.05	0.12 ± 0.00	11.74	6.67 ± 0.21	1.00 ± 0.01	7.93 (13.91)	0.78	19.59 ± 0.13	60.29 ± 0.49	0.00 ± 0.00	0.00 ± 0.00	80.22 (69.01)
	White Organic seeds	7.12 ± 0.03	3.43 ± 0.02	0.11 ± 0.00	11.26	6.33 ± 0.04	0.95 ± 0.01	7.52 (14.83)	0.78	19.95 ± 0.05	60.84 ± 0.14	0.00 ± 0.00	0.00 ± 0.00	81.11 (78.43)
Pumpkin seeds (*Cucurbita pepo*)	Seeds	16.22 ± 0.40	8.28 ± 0.25	1.70 ± 0.23	27.71	30.80 ± 0.20	0.00 ± 0.00	31.56 (90.28)	33.20	35.33 ± 0.71	0.36 ± 0.03	2.57 ± 0.17	0.00 ± 0.00	38.51 (5 4.01)
	Organic seeds	11.92 ± 0.13	6.81 ± 0.06	0.06 ± 0.00	20.03	39.54 ± 0.25	0.00 ± 0.00	39.7 (105.05)	29.86	39.11 ± 0.10	0.45 ± 0.17	0.39 ± 0.03	0.00 ± 0.00	39.98(51.87)
Flaxseed (*Linumusitatissimum)*	Brown	5.84 ± 0.01	4.38 ± 0.02	0.10 ± 0.00	11.01	19.20 ± 0.02	0.00 ± 0.00	19.3 (46.52)	0.76	15.47 ± 0.14	53.91 ± 0.35	0.00 ± 0.00	0.05 ± 0.00	69.58 (82.22)
	Organic golden	5.97 ± 0.08	3.43 ± 0.03	0.12 ± 0.00	10.24	16.85 ± 0.17	0.00 ± 0.00	16.94 (37.94)	0.74	13.01 ± 0.06	59.90 ± 0.27	0.00 ± 0.00	0.06 ± 0.00	73.08(80.25)
	Organic brown	6.58 ± 0.01	5.55 ± 0.03	0.11 ± 0.00	12.98	20.73 ± 0.04	0.00 ± 0.00	20.86 (51.33)	0.76	14.25 ± 0.06	51.76 ± 0.18	0.00 ± 0.00	0.00 ± 0.00	66.18 (79.85)
**Triticale** **(*Triticosecale***)	Wholegrain flour	18.54 ± 0.01	1.35 ± 0.21	0.26 ± 0.05	21.42	15.34 ± 0.18	0.00 ± 0.00	15.84 (2.83)	2.77	53.28 ± 2.27	8.40 ± 2.90	0.00 ± 0.00	0.21 ± 0.02	61.96 (5.42)
	Cereal/meal	16.84 ± 0.08	0.93 ± 0.02	0.21 ± 0.02	19.04	14.29 ± 0.14	0.00 ± 0.00	14.74 (2.05)	3.17	57.93 ± 0.3	7.46 ± 0.50	0.00 ± 0.00	0.23 ± 0.01	65.69 (4.48)
	Rolled	16.28 ± 0.10	1.15 ± 0.02	0.37 ± 0.14	18.81	17.19 ± 0.05	0.00 ± 0.00	17.6 (2.05)	3.64	56.74 ± 0.51	6.27 ± 0.49	0.00 ± 0.00	0.10 ± 0.01	63.16 (3.60)

Where: ^1^ cis-9-octadecenoic acid is oleic acid; ^2^ cis,cis-9,12-octadecadienoic acid is linoleic acid; ^3^ cis,cis,cis-9,12,15-octadecatrienoic acid is α-linolenic acid; ^4^ represents % contribution to RNI for polyunsaturated fat [13] males 18 g/d, females 14 g/day, and this has been calculated as average. The percentages for which to calculate grams per day of fat (35% food energy); saturated fat (11% food energy); polyunsaturated fat (6.5% food energy) and monounsaturated fat (13% food energy). ^5^ represents % contribution to RNI for monounsaturated fat [13] males 36 g/d, females 29 g/day, and this has been calculated as average. The percentages for which to calculate grams per day of fat (35% food energy); saturated fat (11% food energy); polyunsaturated fat (6.5% food energy) and monounsaturated fat (13% food energy).

## Data Availability

The original contributions presented in this study are included in the article/Supplementary Materials. Further inquiries can be directed to the corresponding author. For the purpose of open access, the author has applied a CC BY Creative Commons Attribution (CC BY) licence to any Author Accepted Manuscript arising from this submission.

## Supplementary Materials

The following supporting information can be downloaded at: https://www.mdpi.com/article/10.3390/plants15132079/s1, Table S1: Contribution of 100 g milled crops to Reference Nutrient Intake (RNI) (%) for total carbohydrate, total protein, total fat and fiber (as NSP) for red quinoa grain organic, black quinoa grain organic, organic chia seeds, white organic chia seeds, pumpkin seeds, organic pumpkin seeds, brown flaxseed, organic golden flaxseed, organic brown flaxseed, triticale organic, triticale cereal/meal, triticale rolled; Table S2: Contribution of 100 g milled crops to Reference Nutrient Intake (RNI) (%) for amino acids for red quinoa grain organic, black quinoa grain organic, organic chia seeds, white organic chia seeds, pumpkin seeds, organic pumpkin seeds, brown flaxseed, organic golden flaxseed, organic brown flaxseed, triticale organic, triticale cereal/meal, triticale rolled; Table S3: Composition of fatty acids as % of total fat for red quinoa grain organic, black quinoa grain organic, organic chia seeds, white organic chia seeds, pumpkin seeds, organic pumpkin seeds, brown flaxseed, organic golden flaxseed, organic brown flaxseed, triticale organic, triticale cereal/meal, triticale rolled; Table S4: Contribution of 100 g milled crops to Reference Nutrient Intake (RNI) (%) for of fatty acids as % of total fat for red quinoa grain organic, black quinoa grain organic, organic chia seeds, white organic chia seeds, pumpkin seeds, organic pumpkin seeds, brown flaxseed, organic golden flaxseed, organic brown flaxseed, triticale organic, triticale cereal/meal, triticale rolled; Table S5: Percentage of each seed and micronutrient per 100 g in respect to Reference Nutrient Intake (RNI); Table S6: Phytochemicals concentrations in mg/kg ± SD (n = 3) measured in quinoa by LC-MS/MS analysis; Table S7: Phytochemicals concentrations in mg/kg ± SD (n = 3) measured in chia by LC-MS/MS analysis; Table S8: Phytochemicals concentrations in mg/kg ± SD (n = 3) measured in pumpkin by LC-MS/MS analysis; Table S9: Phytochemicals concentrations in mg/kg ± SD (n = 3) measured in flaxseed by LC-MS/MS analysis; Table S10: Phytochemicals concentrations in mg/kg ± SD (n = 3) measured in triticale by LC-MS/MS analysis.

## Abbreviations

The following abbreviations are used in this manuscript:

LC-MS/MS	Liquid Chromatography–Mass Spectrometry/Mass Spectrometry
NCDs	Non-Communicable Diseases
PUFA	Polyunsaturated Fatty Acid
MUFA	Monounsaturated Fatty Acid
SCFA	Short Chain Fatty Acid
FAO	Food Agriculture Organization
ALA	Alpha Linoleic Acid
NSP	Non-Starch Polysaccharide
RNI	Reference Nutrient Intake
ICP-MS	Inductively Coupled Plasma Mass Spectrometry
PCA	Principal Component Analysis
HPLC	High-Performance Liquid Chromatography
UPLC	Ultra Performance Liquid Chromatography
DIAAS	Digestible Indispensable Amino Acid Score
PDCAAS	Protein Digestibility Corrected Amino Acid Score
WHO	World Health Organization
DMSO	Dimethyl Sulfoxide
