# Supplementary material for: Nutritional and Phytochemical Characterization of Commercially Available Chia, Quinoa, Pumpkin Seed, Flaxseed and Triticale Products
Source: Plants (Basel). 2026 Jul 3;15(13):2079. doi: 10.3390/plants15132079 (PMC13363930; doi:10.3390/plants15132079)
Supplement: Supplementary file 1 [file plants-15-02079-s001.zip › supplementary material_after review.pdf]

Supplementary material

Table S1. Contribution of 100g milled crops to Reference Nutrient Intake (RNI) (%) for total carbohydrate, total protein, total fat and fibre (as NSP) for red quinoa grain organic, black quinoa grain organic, organic chia seeds, white organic chia seeds, pumpkin seeds, organic pumpkin seeds, brown flaxseed, organic golden flaxseed, organic brown flaxseed, triticale organic, triticale cereal/meal, triticale rolled

		Total protein (%RNI)[1]		*Total fat (%RNI)*		Fibre*(%RNI)*	Fibre(1.33*NSP)
		56g/d males	*45g/d females*	*97g/d males*	*78g/d females*	30g/d	
Quinoa (*Chenopodium Quinoa*)	Red Quinoa grain organic	22.58	28.10	5.69	7.08	24.41	7.32
	Black Quinoa grain organic	25.45	31.67	6.00	7.46	28.45	8.53
	Quinoa grain	22.40	27.84	6.07	7.55	21.18	6.35
Chia seeds (*Salvia hispanica*)	Organic Chia seeds	45.68	56.85	8.49	35.43	49.59	14.88
	White Organic Chia seeds	42.93	53.43	32.02	39.82	61.20	18.36
Pumpkin seeds (*Cucurbita pepo*)	Pumpkin seeds	51.34	63.89	46.45	57.76	23.34	7.00
	Organic Pumpkin seeds	63.99	79.63	42.97	53.43	23.92	7.18
Flaxseed (*Linum usitatissimum*)	Brown flaxseed	42.00	52.27	39.13	48.67	49.96	14.99
	Organic golden flaxseed	45.87	57.08	36.37	45.23	40.59	12.18
	Organic brown Flaxseed	39.29	48.89	39.96	49.69	37.93	11.38
Triticale (*Triticosecale)*	Triticale whole grain flour	23.47	29.21	2.90	3.60	35.36	10.61
	Triticale cereal / meal	23.07	28.77	2.26	2.81	31.27	9.38
	Triticale rolled	25.19	31.34	1.89	2.35	29.74	8.92

[1]Where RNI: the amount of a nutrient that is enough to ensure that the needs of nearly all the group (97.5%) are being met.Total fibre= 1.33*NSP

Table S2. Contribution of 100g milled crops to Reference Nutrient Intake (RNI) (%) for amino acids for red quinoa grain organic, black quinoa grain organic, organic chia seeds, white organic chia seeds, pumpkin seeds, organic pumpkin seeds, brown flaxseed, organic golden flaxseed, organic brown flaxseed, triticale organic, triticale cereal/meal, triticale rolled.This considering the quantity for each amino acid required (in mg/kg body weight/day) for a man of 75 kg and a woman of 60 kg.

	Phe-Tyr		Meth-Cys		His		Ile		Leu		Lys		Met		Phe		Thr		Val	
	M	W	M	W	M	W	M	W	M	W	M	W	M	W	M	W	M	W	M	W
**Quantity required (in mg/kg/day) Men (75Kg) Women (60Kg)**	**1050**	**840**	**975**	**780**	**600**	**480**	**750**	**600**	**1050**	**840**	**900**	**720**	**975**	**780**	**1050**	**840**	**525**	**420**	**750**	**600**
Red Quinoa grain organic	61.76	77.20	47.86	59.82	45.60	57.00	48.56	60.70	60.34	75.42	63.29	79.11	24.96	31.20	35.62	44.53	69.03	86.29	61.22	76.53
Black Quinoa grain organic	61.58	76.97	103.74	129.67	45.32	56.64	48.45	60.57	59.73	74.67	63.58	79.47	58.08	72.60	35.79	44.74	70.14	87.67	62.23	77.79
Quinoa grain	53.54	66.92	56.72	70.91	38.02	47.53	41.03	51.29	50.25	62.81	53.27	66.59	29.58	36.97	29.64	37.05	59.25	74.06	52.98	66.22
Organic Chia seeds	151.19	188.99	48.65	60.82	83.92	104.90	93.46	116.83	124.98	156.22	103.92	129.91	25.87	32.34	92.41	115.51	137.49	171.86	125.44	156.81
White Organic Chiaseeds	88.23	110.29	163.24	204.06	49.36	61.70	56.00	70.00	74.00	92.50	66.08	82.60	96.38	120.48	53.04	66.30	81.96	102.45	76.35	95.43
Pumpkin seeds	210.07	262.58	101.92	127.40	99.87	124.84	140.53	175.66	189.68	237.10	116.86	146.07	73.41	91.77	129.97	162.46	156.95	196.19	185.98	232.47
Organic Pumpkin seeds	213.88	267.34	137.90	172.37	100.72	125.90	138.51	173.13	192.06	240.08	126.49	158.11	81.57	101.96	127.68	159.59	161.69	202.11	183.95	229.93
Brown flaxseed	115.26	144.08	99.41	124.26	58.33	72.91	95.50	119.37	98.62	123.27	77.89	97.37	42.07	52.59	74.98	93.73	127.80	159.75	119.87	149.84
Organic golden flaxseed	123.75	154.69	58.17	72.71	62.72	78.40	107.31	134.14	107.60	134.50	86.08	107.61	25.35	31.69	81.08	101.35	136.22	170.28	132.74	165.93
Organic brown Flaxseed	106.31	132.88	102.27	127.84	54.83	68.54	90.18	112.72	94.56	118.20	75.79	94.73	43.39	54.23	68.61	85.77	122.44	153.05	115.19	143.98
Triticale wholegrain flour	73.46	91.83	56.17	70.21	37.91	47.38	45.99	57.49	65.23	81.53	37.58	46.97	23.04	28.80	45.32	56.65	64.08	80.10	64.14	80.18
Triticale cereal/meal	70.12	87.65	25.68	32.10	38.99	48.74	48.86	61.08	67.81	84.76	39.16	48.95	10.70	13.37	46.14	57.68	64.53	80.66	67.72	84.65
Triticale rolled	80.41	100.51	56.06	70.08	29.89	37.37	58.03	72.54	80.28	100.35	47.85	59.82	22.83	28.54	51.32	64.15	72.14	90.17	79.11	98.89

Table S3.Composition of fatty acids as % of total fat for red quinoa grain organic, black quinoa grain organic, organic chia seeds, white organic chia seeds, pumpkin seeds, organic pumpkin seeds, brown flaxseed, organic golden flaxseed, organic brown flaxseed, triticale organic, triticale cereal/meal, triticale rolled

Monounsaturated fatty acids

		trans-9-hexadecenoic acid	cis-9-hexadecenoic acid	trans-9-octadecenoic acid	cis-9-octadecenoic acid	cis-11-octadecenoic acid	cis-15-tetracosenoic acid	total
Quinoa *(Chenopodium Quinoa)*	Red Quinoa grain organic	0.05± 0.01	0.14± 0.00	0.00± 0.00	30.30± 0.04	0.00± 0.00	0.18± 0.02	30.67
	Black Quinoa grain organic	0.06± 0.00	0.26± 0.04	0.00± 0.00	26.31± 0.17	0.00± 0.00	0.16± 0.00	26.79
	Quinoa grain	0.05± 0.01	0.14± 0.00	0.00± 0.00	30.30± 0.04	0.00± 0.00	0.18± 0.02	30.67
Chia seeds *(Salvia hispanica)*	Organic Chia seeds	0.01± 0.01	0.25± 0.01	0.00± 0.00	6.67± 0.21	1.00± 0.01	0.00± 0.00	7.93
	White Organic Chia seeds	0.00± 0.00	0.24± 0.00	0.00± 0.00	6.33± 0.04	0.95± 0.01	0.00± 0.00	7.52
Pumpkin *(Cucurbita pepo)*	Pumpkin seeds	0.00± 0.00	0.10± 0.00	0.28± 0.21	30.80± 0.20	0.00± 0.00	0.03± 0.05	31.56
	Organic Pumpkin seeds	0.00± 0.00	0.11± 0.00	0.01± 0.02	39.54± 0.25	0.00± 0.00	0.00± 0.00	39.70
Flaxseed *(Linum usitatissimum)*	Brown flaxseed	0.03± 0.00	0.07± 0.00	0.00± 0.00	19.20± 0.02	0.00± 0.00	0.00± 0.00	19.30
	Organic golden flaxseed	0.03± 0.01	0.06± 0.01	0.00± 0.00	16.85± 0.17	0.00± 0.00	0.00± 0.00	16.94
	Organic brown Flaxseed	0.02± 0.02	0.11± 0.00	0.00± 0.00	20.73± 0.04	0.00± 0.00	0.00± 0.00	20.86
Triticale *(Triticosecale)*	Triticale wholegrain flour	0.15± 0.01	0.16± 0.02	0.00± 0.00	15.34± 0.17	0.00± 0.00	0.19± 0.05	15.84
	Triticale cereal/meal	0.13± 0.01	0.14± 0.01	0.00± 0.00	14.29± 0.14	0.00± 0.00	0.18± 0.02	14.74
	Triticale rolled	0.14± 0.01	0.12± 0.01	0.00± 0.00	17.19± 0.05	0.00± 0.00	0.15± 0.00	17.60

Saturated Fatty Acids

		tetradecanoic acid	pentadecanoic acid	hexadecanoic acid	heptadecanoic acid	octadecanoic acid	eicosanoic acid	docosanoic acid	tricosanoic acid	tetracosanoic acid	Total
Quinoa (*ChenopodiumQuinoa*)	Red Quinoa grain organic	0.14± 0.01	0.03± 0.01	9.75± 0.06	0.29± 0.03	1.02± 0.07	0.60± 0.01	0.00± 0.00	0.07± 0.00	0.48± 0.00	12.38
	Black Quinoa grain organic	0.23± 0.03	0.06± 0.01	10.77 ±0.17	0.46± 0.01	1.18± 0.11	0.51± 0.01	0.00± 0.00	0.10± 0.00	0.33± 0.01	13.64
	Quinoa grain	0.14± 0.01	0.03± 0.01	9.75± 0.06	0.29± 0.03	1.02± 0.07	0.60± 0.01	0.00± 0.00	0.07± 0.00	0.48± 0.00	12.38
Chia (*Salvia hispanica*)	Organic Chia seeds	0.05± 0.01	0.03± 0.01	7.55± 0.11	0.54± 0.03	3.35± 0.05	0.00± 0.00	0.10± 0.01	0.00± 0.00	0.12± 0.00	11.74
	White Organic Chia seeds	0.04± 0.01	0.02± 0.00	7.12± 0.03	0.45± 0.02	3.43± 0.02	0.00± 0.00	0.09± 0.00	0.00± 0.00	0.11± 0.00	11.26
Pumpkin (*Cucurbita pepo*)	Pumpkin seeds	0.16± 0.01	0.02± 0.01	16.22 ±0.41	0.62± 0.11	8.28± 0.25	0.53± 0.02	0.18± 0.02	0.00± 0.00	1.70± 0.23	27.71
	Organic Pumpkin seeds	0.12± 0.01	0.02± 0.00	11.92 ±0.13	0.50± 0.01	6.81± 0.06	0.45± 0.01	0.15± 0.03	0.00± 0.00	0.06± 0.01	20.03
Flaxseed (*Linum usitatissimum*)	Brown flaxseed	0.05± 0.00	0.02± 0.00	5.84± 0.01	0.48± 0.01	4.38± 0.02	0.00± 0.00	0.14± 0.00	0.00± 0.00	0.10± 0.00	11.01
	Organic golden flaxseed	0.06± 0.01	0.03± 0.01	5.97± 0.08	0.49± 0.02	3.43± 0.04	0.00± 0.00	0.14± 0.00	0.00± 0.00	0.12± 0.00	10.24
	Organic brown Flaxseed	0.06± 0.00	0.02± 0.00	6.58± 0.02	0.49± 0.00	5.55± 0.03	0.00± 0.00	0.17± 0.00	0.00± 0.00	0.11± 0.00	12.98
Triticale (*Triticosecale*)	Triticale whole grain flour	0.17± 0.02	0.17± 0.01	18.54 ±0.75	0.56± 0.09	1.35± 0.21	0.16± 0.01	0.21± 0.04	0.00± 0.00	0.26± 0.04	21.42
	Triticale cereal/ meal	0.15± 0.02	0.15± 0.01	16.84 ±0.08	0.41± 0.05	0.93± 0.02	0.14± 0.00	0.21± 0.01	0.00± 0.00	0.21± 0.02	19.04
	Triticale rolled	0.13± 0.01	0.13± 0.00	16.28 ±0.10	0.42± 0.04	1.15± 0.02	0.14± 0.00	0.19± 0.01	0.00± 0.00	0.37± 0.14	18.81

Polyunsaturated Fatty Acids

		cis,cis-9,12-octadecadienoic acid	cis,cis,cis-6,9,12-octadecatrienoic acid	cis,cis,cis-9,12,15-octadecatrienoic acid	trans,trans-9,11-octadecadienoic acid	cis,cis-11,14-eicosadienoic acid	cis,cis,cis-8,11,14-eicosatrienoic acid	cis,cis,cis-11,14,17-eicosatrienoic acid	cis,cis,cis,cis-5,8,11,14-eicosatetraenoic	cis,cis-13,16-docosadienoic acid	Total
Quinoa *(Chenopodium Quinoa)*	Red Quinoa grain organic	45.77± 0.09	0.00±0.09	8.44± 0.09	0.00±0.09	0.10± 0.00	0.85± 0.01	1.38± 0.01	0.06±0.00	0.00± 0.00	56.60
	Black Quinoa grain organic	50.29± 0.33	0.00±0.33	6.37± 0.15	0.01±0.33	0.12± 0.00	0.72± 0.04	1.49± 0.00	0.06±0.00	0.00± 0.00	59.06
	Quinoa grain	45.77± 0.09	0.00±0.09	8.44± 0.09	0.00±0.09	0.10± 0.00	0.85± 0.01	1.38± 0.01	0.06±0.00	0.00± 0.00	56.6
Chia seeds *(Salvia hispanica)*	Organic Chia seeds	19.59± 0.13	0.32±0.13	60.29± 0.49	0.00±0.13	0.00± 0.00	0.00± 0.00	0.00± 0.00	0.02±0.04	0.00± 0.00	80.22
	White Organic Chia seeds	19.95± 0.05	0.32±0.05	60.84± 0.14	0.00±0.05	0.00± 0.00	0.00± 0.00	0.00± 0.00	0.00±0.00	0.00± 0.00	81.11
Pumpkin seeds *(Cucurbita pepo)*	Pumpkin seeds	35.33± 0.72	0.00±0.72	0.36± 0.03	2.57±0.72	0.16± 0.03	0.00± 0.00	0.00± 0.00	0.00±0.00	0.09±0.01	38.51
	Organic Pumpkin seeds	39.11± 0.10	0.00±0.1	0.45± 0.17	0.39±0.10	0.00± 0.00	0.03± 0.05	0.00± 0.00	0.00±0.00	0.00±0.00	39.98
Flaxseed *(Linum usitatissimum)*	Brown flaxseed	15.47± 0.15	0.15±0.15	53.91± 0.35	0.00±0.15	0.00± 0.00	0.00± 0.00	0.05± 0.00	0.00±0.00	0.00± 0.00	69.58
	Organic golden flaxseed	13.01± 0.06	0.11±0.06	59.90± 0.27	0.00±0.06	0.00± 0.00	0.00± 0.00	0.06± 0.00	0.00±0.00	0.00± 0.00	73.08
	Organic brown Flaxseed	14.25± 0.06	0.17±0.06	51.76± 0.18	0.00±0.06	0.00± 0.00	0.00± 0.00	0.00± 0.00	0.00±0.00	0.00± 0.00	66.18
Triticale *(Triticosecale)*	Triticale whole grain flour	53.28± 2.27	0.00±2.27	8.40± 2.89	0.00±2.27	0.00± 0.00	0.02± 0.02	0.21± 0.02	0.05±0.04	0.00± 0.00	61.96
	Triticale cereal/meal	57.93± 0.38	0.00±0.38	7.46± 0.50	0.00±0.38	0.00± 0.00	0.05± 0.00	0.23± 0.01	0.02±0.03	0.00±0.00	65.69
	Triticale rolled	56.74± 0.51	0.00±0.51	6.27± 0.49	0.00±0.51	0.00± 0.00	0.05± 0.01	0.10± 0.01	0.00±0.00	0.00±0.00	63.16

Table S4. Contribution of 100g milled crops to Reference Nutrient Intake (RNI) (%) for of fatty acids as % of total fat for red quinoa grain organic, black quinoa grain organic, organic chia seeds, white organic chia seeds, pumpkin seeds, organic pumpkin seeds, brown flaxseed, organic golden flaxseed, organic brown flaxseed, triticale organic, triticale cereal/meal, triticale rolled

Samples		TotalFat	Saturated (SFA)		Monoounsaturated (MUFA)		Contributionto RNI		Polyunsaturated (PUFA)		Contribution to RNI	
			Total SFA	SFA *Totalfat	Total MUFA	MUFA *Totalfat	Male	Female	Total PUFA	PUFA * Totalfat	Male	Female
Quinoa (*Chenopodium Quinoa)*	Red Quinoa grain organic	5.80	0.12	0.72	0.31	1.78	9.88	12.71	0.57	3.28	9.12	11.320
	Black Quinoa grain organic	6.33	0.14	0.86	0.27	1.70	9.42	12.11	0.59	3.74	10.38	12.891
	Quinoa grain	6.23	0.12	0.77	0.31	1.91	10.62	13.65	0.57	3.53	9.79	12.159
Chia *(Salvia hispanica)*	Organic Chia seeds	25.33	0.12	2.97	0.08	2.01	11.16	14.35	0.80	20.32	56.44	70.068
	White Organic Chia seeds	26.10	0.11	2.94	0.08	1.96	10.90	14.02	0.81	21.17	58.80	72.999
Pumpkin (*Cucurbita pepo)*	Pumpkin seeds	40.97	0.28	11.35	0.32	12.93	71.83	92.36	0.39	15.78	43.83	54.405
	Organic Pumpkin seeds	43.76	0.20	8.77	0.40	17.37	96.52	124.09	0.40	17.50	48.60	60.328
Flaxseed (*Linum usitatissimum)*	Brown flaxseed	37.50	0.11	4.13	0.19	7.24	40.21	51.70	0.70	26.09	72.48	89.974
	Organic golden flaxseed	36.98	0.10	3.79	0.17	6.26	34.80	44.75	0.73	27.02	75.07	93.190
	Organic brown Flaxseed	39.97	0.13	5.19	0.21	8.34	46.32	59.56	0.66	26.45	73.48	91.214
Triticale (*Triticosecale)*	Triticale wholegrain flour	3.03	0.21	0.65	0.16	0.48	2.67	3.43	0.62	1.88	5.21	6.474
	Triticale cereal/meal	2.68	0.19	0.51	0.15	0.40	2.19	2.82	0.66	1.76	4.89	6.071
	Triticale rolled	1.92	0.19	0.36	0.18	0.34	1.88	2.41	0.63	1.21	3.37	4.182

Table S5. Percentage of each seed and micronutrient per 100g in respect to Reference Nutrient Intake (RNI).

	Na %RNI	Mg %RNI	P %RNI	K %RNI	Ca %RNI	Mn %RNI	Zn %RNI	Fe %RNI
**Black Quinoa grain organic**	1.35	69.05	80.98	25.73	10.89	259.44	37.20	50.51
**Brown Flaxseed**	5.63	138.99	128.82	26.77	37.27	194.84	73.94	62.29
**Organic brown Flaxseed**	**8.84**	142.84	119.62	22.03	35.46	214.19	67.96	63.45
**Organic Chia seeds**	5.46	137.12	151.23	24.10	**80.67**	279.11	70.57	**77.28**
**Organic golden Flaxseed**	6.97	146.18	107.39	23.12	46.55	189.81	76.33	**77.34**
**Pumpkin seeds**	3.28	188.26	194.42	24.94	12.11	308.03	53.27	65.62
**Organic Pumpkin seeds**	3.42	**206.38**	**223.00**	**27.00**	12.19	**333.93**	**82.10**	72.13
**Quinoa grain**	7.67	66.27	81.22	17.32	5.31	117.01	32.89	42.53
**Red Quinoa grain organic**	9.28	69.52	87.65	21.44	7.03	142.99	33.70	47.51
**Triticale cereal/meal**	1.50	57.18	83.12	16.46	4.18	333.58	35.02	33.55
**Triticale wholegrain flour**	2.71	52.32	75.66	15.59	6.69	313.01	41.99	35.51
**Triticale rolled**	1.47	52.42	69.52	15.64	5.60	233.34	47.23	28.65
